# Obesity and Mental Health in Childhood and Adolescence: A Scoping Review of Recent Scientific Evidence

**DOI:** 10.3390/children12111512

**Published:** 2025-11-07

**Authors:** María Morales-Suárez-Varela, Esther López-García, Isabel Peraita-Costa, José Manuel Pérez Puente, Agustín Llopis-Morales, Agustín Llopis-Gonzalez, Pilar Guallar-Castillón

**Affiliations:** 1 Research Group on Social and Nutritional Epidemiology, Pharmacoepidemiology, and Public Health, Department of Preventive Medicine and Public Health, Food Science, Toxicology, and Legal Medicine, Faculty of Pharmacy and Food Sciences, Universitat de València, Av. Vicent Andrés Estellés 22, 46100 Burjassot, Spain; isabel.peraita@uv.es (I.P.-C.); jopepuen@alumni.uv.es (J.M.P.P.); allomo@alumni.uv.es (A.L.-M.); agustin.llopis@uv.es (A.L.-G.); 2 Biomedical Research Network Center for Epidemiology and Public Health (CIBERESP), Carlos III Health Institute, Av. Monforte de Lemos 3-5, Pavilion 11, Floor 0, 28029 Madrid, Spain; esther.lopez@uam.es (E.L.-G.); mpilar.guallar@uam.es (P.G.-C.); 3 Department of Preventive Medicine and Public Health, Faculty of Medicine, Autonomous University of Madrid, C/Arzobispo Morcillo, 4, 28029 Madrid, Spain; 4 Madrid Institute for Advanced Studies—Research Institute on Food & Health Sciences (IMDEA-Food), Campus of International Excellence of the Autonomous University of Madrid and Spanish National Research Council (CEI UAM + CSIC). Crta. de Cantoblanco 8, 28049 Madrid, Spain

**Keywords:** obesity, mental health, adolescence, depression, anthropometry

## Abstract

**Highlights:**

**What are the main findings?**

- The evidence supports the existence of a relationship between obesity and mood disorders, especially depression,
- The relationship is multifactorial, bidirectional, and influenced by psychosocial, biological, and environmental factors.

**What is the implication of the main finding?**

- There is a need for early and comprehensive assessments, including not only weight and nutritional parameters but also emotional status.
- The evidence underscores the need for multidisciplinary interventions that integrate nutritional, psychological, and behavioral dimensions.

**Abstract:**

**Background:** Child and adolescent obesity represent an increasing public health concern due to their physical consequences and impact on mental health. Recent studies have shown a significant association between obesity and depressive disorders during childhood and adolescence. The primary aim of this review was to analyze scientific evidence on the relationship between obesity and mental health in children and adolescents, with a particular focus on depressive symptoms and the use of anthropometric indicators. Secondary objectives included identifying the most common assessment tools, describing treatment approaches, and exploring mediating factors. **Methods:** A scoping literature review was conducted. The PubMed, Cochrane, and SciELO databases were searched for records published between 2015 and 2025 that met the inclusion criteria. **Results:** The 24 studies identified revealed a significant association between obesity and depressive symptoms, although considerable methodological heterogeneity was noted. Some studies reported a direct relationship with BMI, whereas others emphasized mediating factors such as body image perception and self-esteem. Cognitive–behavioral interventions and integrated programs showed both physical and psychological benefits. **Conclusions:** The relationship between child and adolescent obesity and mental health is complex and multifactorial. Findings support the development of multidisciplinary interventions that simultaneously address nutritional and psychological aspects.

## 1. Introduction

Childhood and adolescent obesity is one of the major public health concerns of the 21st century. Globally, the number of children and adolescents aged 5–19 years who are overweight or obese has quadrupled since 1975, reaching more than 390 million in 2022, according to the World Health Organization (WHO) [1]. This exponential rise has been observed in both developed and developing countries, establishing obesity as a global public health challenge.

In Spain, prevalence has also increased considerably. According to the National Health Survey (ENS), the prevalence of obesity among adults was 7.7% in 1987, doubled to 13.6% in 2001, and reached 15.6% in 2006 [2]. Currently (2025), it is estimated that approximately 1 in 5 adults and 1 in 10 children and adolescents in Spain are obese [3].

Adolescent obesity often continues from childhood obesity [4], reflecting the persistence of biological, environmental, and behavioral factors throughout development. Beyond aesthetic or social implications, this condition entails serious short- and long-term risks for both physical and mental health [5]. Understanding how childhood obesity translates into adolescent obesity is crucial for designing early and effective interventions.

Obesity during childhood and adolescence not only carries physical consequences—such as hypertension, type 2 diabetes, or musculoskeletal disorders—but it can also significantly affect cognitive function [6,7,8] through an oxidative stress mechanism [9,10]. In societies where thinness is equated with attractiveness, being overweight or obese can have important psychosocial repercussions and these children and adolescents frequently face social stigma, often being perceived as responsible for their condition due to an alleged lack of discipline or willpower [11]. This stigma can negatively affect their mental health and emotional well-being.

The economic and social burden of obesity is equally alarming. In the United States alone, obesity-related consequences account for annual costs exceeding USD 100 billion and are associated with high rates of premature mortality [12].

Adolescence is a period characterized by intense physical, hormonal, and psychosocial changes, increasing vulnerability to mood disorders. Factors such as body image perception, family or peer pressures, and bullying may critically influence psychological well-being. The American Psychiatric Association has highlighted the close relationship between obesity, stigma, and mood disorders during this stage [13].

Depression is among the most prevalent mental health disorders during adolescence, with significant repercussions on academic, social, and personal development. The relationship between obesity and depression is bidirectional: obesity can contribute to depressive symptoms due to stigma, social isolation, or low self-esteem, while depression may lead to unhealthy behaviors—such as sedentarism or disordered eating—that increase the risk of weight gain [14,15,16].

Anthropometry plays a fundamental role in assessing growth, development, and nutritional status. Tools such as BMI, waist circumference, and waist-to-height ratio allow for the identification of risk patterns and support preventive and therapeutic strategies. For adults, overweight is defined as a BMI between 25 and 29.9 kg/m^2^, and obesity as BMI ≥ 30 kg/m^2^. For children and adolescents aged 5–19 years, WHO defines overweight as BMI-for-age > 1 SD above the median, and obesity as BMI-for-age > 2 SD above the median (Table 1). It must be noted that in 2000, the WHO Western Pacific Region introduced the Asia-Pacific BMI classification for Asian adults [17,18,19] and this classification uses a lower BMI cutoff (overweight is defined as a BMI ≥ 23 kg/m^2^ and obesity is defined as a BMI ≥ 27.5 kg/m^2^) than the conventional WHO BMI classification which is applied across all races. Comparatively, an Asian child is considered overweight if their BMI falls between the 85th and 95th percentile within their national standards for their age and sex and obese if it is at or above the ≥95th percentile.

Addressing this public health challenge requires a comprehensive approach that takes into account the biological, psychological, and social factors involved. Understanding the relationship between anthropometric indicators and psychological health during adolescence is essential for designing more effective prevention strategies, reducing the burden of disease in later stages, and promoting comprehensive development during a crucial phase of life.

In recent years, research on the consequences of obesity for mental health has grown substantially, highlighting the need to better understand the complex interplay between obesity and mental health in adolescence. The objective of this review is to provide an updated synthesis of the existing evidence on the relationship between obesity and mental health in children and adolescents, with special attention given to the presence of depressive symptoms and the use of anthropometric indicators in the assessment of this association.

## 2. Methods

### 2.1. Design

This study was conducted as a scoping review of scientific literature, taking the PRISMA-ScR (Preferred Reporting Items for Systematic Reviews and Meta-Analyses extension for Scoping Reviews) guidelines [20,21] and the Joanna Briggs Institute guidelines for scoping reviews [22,23,24,25,26] as methodological references to ensure transparency and rigor in the process of searching, selecting, and analyzing the evidence. A formal review protocol has not been registered.

### 2.2. Search Strategy

A comprehensive search was performed in three major scientific databases: PubMed, Cochrane Library, and SciELO. The time frame covered publications from January 2015 to March 2025. To identify relevant studies, combinations of descriptors and Boolean operators were applied:

- (“obesity” OR “overweight” OR “body mass index”)
- AND (“adolescence” OR “youth”)
- AND (“mental health” OR “depression” OR “psychological well-being”).

These terms were selected to capture research focused on the intersection between obesity, adolescence, and mental health outcomes.

### 2.3. Eligibility Criteria

The review considered studies that met the following inclusion criteria:

1. Original articles, including observational and intervention studies.
2. Participants aged between 10 and 19 years.
3. Assessment of overweight or obesity through anthropometric indicators such as BMI, waist circumference, or waist-to-height ratio.
4. Evaluation of mental health outcomes, including depressive symptoms, depression, or other psychological variables.
5. Articles published in English or Spanish.

Exclusion criteria were defined to maintain focus and relevance. Studies were excluded if they:

1. Investigated populations with chronic diseases not related to obesity.
2. Focused exclusively on adults or on children under 10 years of age.
3. Were classified as editorials, commentaries, or conference abstracts.

### 2.4. Article Selection and Data Extraction and Synthesis

Article selection and data extraction were performed independently by two reviewers to minimize bias. Any disagreements between reviewers were resolved through discussion and consensus with the inclusion of a third party if no agreement was initially reached.

Information extracted from each study included:

- study design,
- population characteristics,
- anthropometric measures used,
- psychological assessment tools applied,
- main findings, and
- reported limitations.

In accordance with the methodological guidelines used as reference [20,21,22,23,24,25,26], the most relevant information from the included studies is presented in the various tables. Given the heterogeneity of the included studies in terms of design, measures, and outcomes, a narrative synthesis was carried out. This approach allowed for the integration of findings while highlighting common patterns and key differences across studies.

## 3. Results

**Figure 1:**
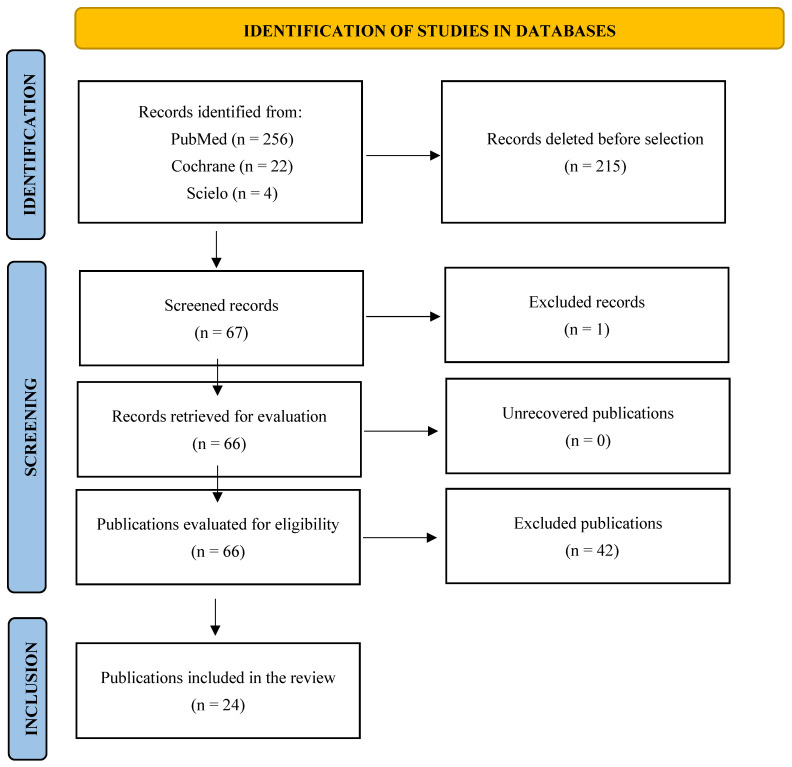
PRISMA-ScR flowchart of the publication selection process.

The initial search yielded 282 references: 256 in PubMed, 22 in the Cochrane Library, and 4 in SciELO. After removing duplicates (*n* = 64), a total of 218 titles and abstracts were screened. Of these, 174 articles were excluded for not meeting the inclusion criteria. The remaining 44 full-text articles were examined in detail. Following this stage, 20 articles were excluded due to methodological limitations or lack of relevance to the objectives of the review. Ultimately, 24 studies were included in the final analysis (Figure 1).

Among them, 13 were observational studies and 11 were intervention studies. Of the 24, 7 were cross-sectional studies and 17 longitudinal studies. Sample sizes ranged widely, from 120 to more than 18,000 adolescents, and the studies were conducted in Europe, North America, Latin America, and Asia.

### 3.1. Observational Studies

The 13 observational studies included show considerable methodological heterogeneity, especially in the instruments used to assess mental health in adolescents. The tools applied cover different domains: depressive symptoms, anxiety, self-esteem, body image, and executive function, all of which are particularly relevant aspects at this stage of development. Among the most widely used [27,28,29,30] scales were the Center for Epidemiologic Studies Depression Scales (CES-D) [31,32], which has been extensively validated in adolescent populations previously and the children’s version CES-DC [33,34].

Another frequently used tool [2,35,36,37] was the Beck Depression Inventory (BDI), used in its BDI-I, BDI I-A and BDI-II versions [38,39,40]. Other scales used in combination for depression and anxiety were the Depression Anxiety and Stress Scale 21 (DASS-21) [41], used by Alsaleem et al., 2021 [42], and the Revised Children’s Anxiety and Depression Scale (RCADS-11) [43], used by Öz & Kıvrak, 2023 [16], which allow for a broader assessment of general psychological distress. Less frequently used but still relevant, the Mood Scale Patient Health Questionnaire (MSPHQ) [44], based on criteria from the Diagnostic and Statistical Manual of Mental Disorders, fourth edition (DSM-IV) [45], was used by Al Dweik et al., 2022 [46], and clinical questionnaires based on the DSM-IV were applied by Roberts & Duong, 2015 [15]. Regarding other psychological dimensions, complementary tools were used, such as Rosenberg’s self-esteem scale [2,16,47], a single self-perception question [48], and the Piers-Harris Children’s Self Concept Scale (PHCSCS) [16,49,50]. Body image was assessed using self-perceived weight [15], a silhouette scale [37], and the Body Shape Questionnaire (BSQ) [2,15,16,29,48,51].

BMI was the most frequently used tool to define obesity. Nevertheless, some studies found that other measures, such as waist circumference and waist-to-height ratio, were more closely associated with psychological outcomes. These measures may better capture fat distribution, which could influence self-perception and mental health.

#### 3.1.1. Direct Association Between BMI and Mental Health

Five studies reported a positive association between BMI and mental health (Table 2).

In a representative sample of adolescents in China, Zhao et al., 2019 [30] identified a significant relationship between overweight/obesity and depressive symptoms, especially in males. Subdimensions such as depressive affect and lack of positive affect were linked to an increased risk of obesity.

In Latin America, Morán et al., 2024 [2] found that adolescents with higher BMI had higher scores on depression, anxiety, body dissatisfaction, and low self-esteem. Similarly, according to age, Ocampo et al., 2017 [36] found that overweight/obese adolescents had an 8.55 times higher risk of presenting depressive symptoms, compared to their normal-weight peers.

In the Middle East, Alsaleem et al., 2021 [42] reported that obesity was associated with an increased risk of anxiety (OR = 1.97) and depression (OR = 1.90). Meanwhile, Al Dweik et al., 2022 [46] also found a positive relationship between BMI and depressive symptoms.

#### 3.1.2. No Direct Association Between BMI and Mental Health

Eight studies found no statistically significant relationship between BMI and mental health (Table 3). However, they all agree on highlighting relevant associations between mental health and psychological factors such as self-esteem, body dissatisfaction, subjective perception of weight, and social anxiety.

In South Korea, although the study by Lim et al., 2016 [48] found no direct association between BMI and depression in the total sample, a significant relationship was observed between depressive symptoms, low self-esteem, and negative body perception.

Also in Asia, Wang et al., 2020 [37] found a bidirectional relationship between body dissatisfaction and depression in overweight or obese adolescents. It was found that body dissatisfaction predicted future depressive symptoms and vice versa, suggesting that the emotional effects of excess weight depend more on self-perception (assessed using a silhouette scale) than on target weight.

Although obese adolescents showed significantly higher levels of depression in the study by Öz & Kıvrak, 2023 [16], no direct relationship was found between BMI and depressive symptoms within the obese group. Nor were there any significant differences by gender in the psychological variables analyzed.

In France, Carriere et al., 2019 [35] identified that 41.7% of overweight or obese adolescents had eating disorders (EDs), particularly binge eating disorder (BED), which was associated with both higher BMI and more severe depressive symptoms. However, no direct relationship was found between BMI and depression; both variables were independently associated with EDs.

In the United States, the study by Roberts & Duong, 2015 [15] concluded that negative body image acted as a key mediator between obesity and depressive symptoms. It was found that subjective weight perception fully explained the association between obesity and depression, indicating that the psychological impact is more related to how adolescents perceive themselves than to their actual weight. In addition, gender differences were identified: in girls, negative self-perception had a stronger mediating effect than in boys.

The study by Lopez et al. 2021 [29], also in the United States, found no significant relationship between BMI and depressive symptoms in overweight or obese adolescents. However, adolescents with more depressive symptoms had more cognitive difficulties, especially in executive functions such as organization, emotional control, and decision-making. In addition, more than 25% of participants had depressive symptoms at the start of the study, indicating a high prevalence in this population.

In African American and Hispanic adolescents, the study by Eitle & Eitle, 2018 [28] showed that self-perception of being overweight, rather than objective overweight, was associated with depressive symptoms.

In Brazil, the study by Antunes Lima et al., 2020 [27] determined that social anxiety was the psychological variable most closely related to obesity, as assessed by waist circumference. It was observed that adolescents with obesity experienced greater social avoidance and fear of negative evaluation than depressive symptoms.

In summary, the available evidence indicates that, although a direct association between BMI and depression is not always identified, intermediate variables such as self-esteem, body dissatisfaction, subjective weight perception, social anxiety, and negative body image play an essential mediating role in the relationship between obesity and mental health. However, the magnitude and nature of the relationship vary depending on the sociocultural context, gender, age, and assessment instruments used, underscoring the need for individualized, interdisciplinary, and culturally sensitive approaches. Understanding these factors is essential for designing more effective psychological interventions targeting this vulnerable population.

### 3.2. Intervention Studies

The articles included in this review that report on intervention studies show considerable heterogeneity in the methods used to assess mental health, the interventions applied, and their respective outcomes. In terms of the instruments used, various validated scales that assess symptoms of depression, anxiety, stress, self-esteem, and eating disorders (EDs) were used.

For the assessment of depression, tools such as the Children’s Depression Inventory (CDI and CDI-2) [31,52] were used [53,54,55], along with the BDI-II [56] and the DASS-21 [57]. To measure stress and general well-being, the Perceived Stress Scale (PSS) [58] was used [56,59] and the Rosenberg self-esteem scale [60,61]. In terms of the assessment of EDs, the Eating Disorder Examination Questionnaire (EDE-Q) [62,63,64], the Binge-Eating Scale (BES) [55,65], and the Children’s Eating Attitudes Test (ChEAT) [57,66] were used.

Most studies have a longitudinal design, which allows for the observation of changes in clinical and psychological variables over time. This approach promotes a better understanding of the impact of interventions on mental health and body weight in adolescents with obesity. The prospective longitudinal studies [53,54,56,57,59,61,63,64,67,68] stand out for their methodological control. In contrast, despite its methodological limitations, the only retrospective study identified [60], provides relevant insights into the implementation of programs in real-world settings.

With regard to intervention, nine of the eleven [53,54,56,57,59,60,63,67,68] studies reviewed applied behavioral interventions focused on psychological aspects of the patient. Others opted for strategies based on dietary restriction [55], and one resorted to bariatric surgery (gastric bypass) [61] as a treatment for severe obesity. Overall, the studies analyzed show that the most effective interventions integrate psychoeducational, behavioral, and social components tailored to the individual characteristics of adolescents.

#### 3.2.1. Cognitive Behavioral Therapies

Although most studies use cognitive behavioral therapies (CBT) focused on the psychological aspect of patients, the results in terms of body weight loss and improvements in mental health and depressive symptoms show significant variability (Table 4).

The study by Gulley et al., 2019 [53], despite not finding a significant direct relationship between depression and BMI before treatment, considered previous evidence indicating that depression can impair metabolic health. In this study, CBT therapy reduced depressive symptoms, but this improvement in mental health did not translate into an overall reduction in BMI in all patients; however, an improvement in fasting insulin levels, an important marker of risk for type 2 diabetes, was observed. In fact, the reduction in BMI was only evident in patients with severe depression prior to treatment.

Naar-King et al., 2016 [67] applied a treatment based on motivational interviews and behavior modification, comparing different formats (at home vs. in the office, and with incentives vs. skills training). The adolescents also received dietary support and clinical monitoring. The results showed a significant reduction in overweight, although without a significant impact on depressive symptoms, which contrasts with other studies that did report mood improvement associated with weight loss.

The SHINE program, led by Nobles et al., 2016 [60], is an intensive community-based intervention for adolescents with severe obesity that combines physical activity, nutrition education, and psychological support. Developed in three phases over up to 12 months, it showed a significant reduction in BMI and notable improvements in mental health: a 49.9% decrease in anxiety, a 54% decrease in depressive symptoms, and a 38% increase in self-esteem after the active phase. The intervention had high retention (94.8%), and no significant differences were found between genders, suggesting similar effectiveness for boys and girls.

The APOLO-Teens study, conducted by Ramalho et al., 2020 [57], was a 12-week CBT intervention conducted via Facebook, focused on promoting healthy lifestyle habits and improving mental health in overweight adolescents. Participants had high levels of depression, anxiety, stress, and impulsivity at baseline, which were negatively related to quality of life. Gender differences were identified: girls showed greater psychological distress, dysfunctional eating behaviors, greater impulsivity, less physical activity at school, and a worse perception of physical and psychosocial quality of life compared to boys.

Similarly, Tronieri et al., 2019 [56] evaluated an intervention based on acceptance and commitment therapy which, after 16 weeks, produced a slight reduction in BMI and a significant improvement in mood and quality of life, mainly in the social sphere. The intervention included sessions on emotional regulation, mindfulness, and decision-making based on personal values, as well as dietary recommendations, physical activity, and parental involvement.

Tulloch et al., 2020 [59] evaluated a behavioral intervention in overweight/obese adolescents focused on physical and psychological parameters. Improvements in physical fitness (six-minute walk test) were observed, but no significant association was found between BMI reduction and improvement in mental health, such as depressive symptoms, perceived stress, self-efficacy, or outcome expectations.

Darling et al., 2021 [63] analyzed an outpatient behavioral group therapy for overweight or obese adolescents, focusing on nutrition education and increased physical activity to modify behaviors. A significant improvement in depressive symptoms and dietary self-control was observed, especially in those with more severe symptoms at baseline, who also achieved a greater reduction in BMI.

Hoare et al., 2016 [68] evaluated a school-based intervention aimed at improving diet and physical activity in adolescents. Although there were no significant direct effects on mental health, it was observed that in males, physical activity was associated with fewer depressive symptoms, while in females, increased fast food consumption was associated with a higher risk of depression.

Finally, the study by Lemstra & Rogers, 2022 [54], “The Healthy Kids Initiative,” implemented an intervention that included five weekly exercise sessions, one nutrition session, and one group CBT session. The intervention did not aim at weight loss, nor was body weight monitored, seeking to preserve mental health. Depressive symptoms decreased significantly, from 79.2% to 64.7% after the intervention, remaining at 67.7% after one year, with a greater effect in adolescents under 14 years of age. Improvements in self-esteem and quality of life were also observed, especially in those who improved their diet.

#### 3.2.2. Restrictive Therapies

Although most studies include nutritional education as an important component of treatment, only one of them considers diet to be the central focus of the intervention, which represents a significant difference from the rest of the articles reviewed (Table 5).

The Fast Track to Health therapy, used by Jebeile et al., 2024 [55], consisted of a restrictive diet for 52 weeks, in which two groups with different types of energy restriction were compared: intermittent energy restriction, applied three days a week, and continuous energy restriction. Both groups started with a very low-calorie diet and were then assigned to one of the two regimens, also receiving psychological support throughout the process.

Both groups showed significant improvements in levels of depression and concern about body weight, as well as a reduction in BMI. In the first four weeks, decreases in eating disorders such as binge eating episodes were observed, although these improvements were maintained only in the intermittent energy restriction group during the 52 weeks. It is important to note that 12.1% of adolescents required additional psychological support due to mental health symptoms during the study. These results were sustained throughout the year, providing evidence of long-term effects superior to those reported in other studies.

#### 3.2.3. Surgical Therapies

Among the articles reviewed, only one addresses surgical treatment: laparoscopic gastric bypass [61] (Table 6). Before undergoing surgery, all patients had received at least one year of conventional treatment at a pediatric clinic specializing in obesity.

At the start of the study, the average BMI was 45.6 kg/m^2^, and after two years of follow-up, it had significantly decreased to 30.1 kg/m^2^. In fact, 50% of patients no longer met the criteria for obesity at the end of the follow-up period.

In addition to weight reduction, significant improvements in mental health were observed during the first year, which remained stable in the second year. There was a decrease in symptoms of depression, anxiety, and irritability, as well as an increase in self-esteem and overall mood. There was also a reduction in social and emotional difficulties related to excess weight.

However, 19% of adolescents continued to show clinically relevant depressive symptoms two years after surgery, highlighting the need for prolonged psychological follow-up. Girls showed greater improvement in depressive symptoms after surgery compared to boys, especially those with high levels of depression at the start of treatment.

In summary, the available evidence suggests that a comprehensive approach to combating obesity, one that considers not only weight reduction but also mental health improvement particularly through cognitive–behavioral therapy, is essential to achieving sustainable results and improving the quality of life of this vulnerable population.

## 4. Discussion

This review confirms that the relationship between obesity and depression in childhood and adolescence is multifactorial, bidirectional, and influenced by psychosocial, biological, and environmental factors.

The results obtained in this review largely coincide with previous findings in the literature, which support the existence of an association between obesity and mood disorders, especially depression [2,15,30]. The review by Hoare et al., 2013 [69] identified various obesogenic risk factors, such as physical inactivity, poor diet, negative body image, and low social support, all of which are related to depressive symptoms, reinforcing the results presented here.

However, the direct relationship between BMI and depression was not uniform across all studies, a phenomenon also described by other authors [16,35]. These authors suggest that intermediate factors, such as weight perception, body dissatisfaction, and self-esteem, mediate this association. This approach is also supported by Whitlock et al., 2005 [70], who emphasize that emotional distress in adolescents with obesity does not depend exclusively on weight, but also on the social environment and self-image.

On the other hand, the review by Mühlig et al., 2015 [71], highlights that the association between obesity and depression may be bidirectional from an early age, a finding that we also find in studies such as those by Wang et al., 2020, [37] and Roberts & Duong, 2015 [15]. Obesity can promote the development of depressive symptoms, while children and adolescents with depression may adopt unhealthy lifestyle behaviors, such as sedentarism, irregular eating patterns, emotional eating, and poor sleep quality, which contribute to weight gain [72,73]. These habits not only perpetuate depressive symptoms but also increase the likelihood of obesity, creating a self-reinforcing cycle between both conditions [74]. This complexity is crucial to understanding the interaction between psychological and physical factors during adolescence, a particularly vulnerable stage of life.

It is also important to consider that children and adolescents with obesity are more vulnerable to social stigma, teasing, and bullying, which often lead to social isolation, reduced self-esteem, and feelings of worthlessness. These psychosocial stressors significantly increase the risk of developing depression [75,76]. Hoare et al., 2013 [69] point out that a hostile social environment exacerbates negative body perception, especially in adolescent girls. Body image dissatisfaction emerges as one of the strongest predictors of depressive symptoms, sometimes outweighing the predictive value of BMI itself [77,78]. This suggests that the perception of one’s own body may be as relevant as objective anthropometric indicators.

From a clinical perspective, these findings underscore the need for comprehensive assessments of children and adolescents with obesity, including not only weight and nutritional parameters but also emotional status. Cognitive–behavioral therapies evaluated in studies such as those by Nobles et al., 2016 [60], Lemstra & Rogers, 2022 [54], and Ramalho et al., 2020 [57] have proven effective in improving both BMI and mental health, positioning themselves as first-line interventions.

Other approaches, such as acceptance and commitment therapy [56] or group programs such as SHINE [60], stand out for their community applicability and good adherence, which facilitates their implementation in clinical, educational, and social contexts for a more accessible and sustainable approach.

It has been observed that the impact of interventions is not homogeneous. For example, Ramalho et al., 2020 [57] showed that girls have greater depressive symptoms and psychological vulnerability, which requires gender-sensitive interventions addressing emotional, behavioral, and relational aspects in addition to body weight.

In preventive medicine, this review reinforces the importance of early detection of depressive symptoms in overweight or obese adolescents, both in primary care and in school settings. Childhood obesity is a strong predictor of obesity in adolescence, especially when habits are not corrected and early intervention does not occur. Evidence shows that weight tends to remain stable in the absence of intervention, with lasting implications for metabolic and mental health (Longitudinal Changes in Weight Status from Childhood and Adolescence to Adulthood [4]. This would facilitate early interventions, improve adherence to weight loss programs, and help prevent the chronicity of psychological distress.

The results also provide evidence to guide public policies aimed at adolescents, proposing the inclusion of psychological interventions in pediatric obesity care units and integrated school programs for physical and emotional well-being, contributing to the prevention of long-term mental health problems.

The evidence underscores the need for multidisciplinary interventions that integrate nutritional, psychological, and behavioral dimensions. The design of multidisciplinary interventions that integrate the emotional component increases therapeutic efficacy, reduces dropout rates, and improves the medium- and long-term prognosis. Treatments that combine nutritional education, promotion of physical activity, and cognitive–behavioral therapy have demonstrated improvements not only in weight control but also in depressive symptoms [69]. These interventions should be personalized and take gender differences into account, as evidence shows greater emotional vulnerability in girls [2,15,30]. This integrated approach appears more effective than strategies focused exclusively on weight reduction, as it addresses both the physical and psychological determinants of health.

In short, obesity in childhood and adolescence cannot be addressed exclusively from a nutritional perspective. Its psychological impact requires a comprehensive, interdisciplinary, and culturally sensitive approach that combines prevention, early diagnosis, psychoeducational intervention, and sustained clinical follow-up. Only then can the emotional and physical burden of obesity on this vulnerable population be reduced.

### 4.1. Strengths and Limitations

Despite the growing body of literature, several limitations should be considered. The studies included show considerable methodological heterogeneity, both in terms of design (cross-sectional, prospective or retrospective longitudinal) and the tools used to assess mental health. This diversity makes it difficult to compare results directly, although it enriches our overall understanding of the phenomenon.

The predominance of cross-sectional studies restricts the ability to establish causality [79]. However, the inclusion of longitudinal designs is a strength, as it allows for observation of evolution and the formulation of causal hypotheses, while cross-sectional studies offer valuable information on associations, although without establishing causality. Also, intervention studies often have limited follow-up, making it difficult to assess the sustainability of the observed effects.

Despite these limitations, the evidence allows us to identify relevant trends and formulate solid hypotheses for future research with greater methodological homogeneity and control of confounding variables.

High-quality longitudinal studies are needed to evaluate the bidirectional relationship between anthropometric indicators and mental health, incorporating biomarkers of inflammation and metabolic stress to clarify pathophysiological mechanisms [71].

Furthermore, there is significant heterogeneity in the tools used to evaluate mental health as well as in the anthropometric measures applied which may undermine the robustness of the conclusions extracted. In terms of mental assessment, various validated scales with different psychometric properties were used, which makes direct comparisons difficult and may increase heterogeneity. In addition, most are based on self-administered questionnaires, with little triangulation through clinical interviews or external reports, which may limit diagnostic accuracy.

Mental assessment instruments vary in sensitivity, target population, and approaches (emotional, cognitive, behavioral), which partly explains the variability in results. The literature recommends agreeing on validated tools specifically adapted for the adolescent population [69]. Likewise, the incorporation of qualitative or mixed assessments would allow for a deeper understanding of the subjective experience, an aspect that has been little explored in the studies reviewed.

BMI remains the most widely used tool to classify obesity; however, some research suggests that waist circumference and waist-to-height ratio may provide more precise insights into the relationship with mental health, since they reflect fat distribution more accurately [80,81]. These measures may better explain the association between self-perception of health risk and psychological outcomes.

It is essential that future studies adopt more homogeneous methodologies in terms of instruments and designs to facilitate accurate comparisons. It would also be useful to directly compare the effectiveness of different interventions, ideally in subgroups according to age, sex, and socioeconomic context.

In this review, effect size has not been considered and an analysis on the effect size of each study would be helpful in describing how strong the relationship between obesity and mental health is. Furthermore, effect sizes could then be included in a meta-analysis that combines and synthesizes the results from multiple independent studies on the topic to produce a single, more precise conclusion.

Few studies incorporate contextual variables such as culture, socioeconomic status, or family dynamics, which may play an essential role in the association between obesity and mental health [82].

As noted by Hoare et al., 2016 [68] and Whitlock et al., 2005 [70], there is a need for more research in underrepresented populations, especially in developing countries, where cultural, economic, and health factors can have a significant influence. Finally, it is important to explore the impact of social media, exposure to idealized bodies, and bullying as psychosocial factors that may mediate or catalyze this complex relationship.

### 4.2. Future Directions

Future research should prioritize longitudinal designs using standardized methodologies that allow for the identification of causal mechanisms. Additionally, studies should explore how cultural norms, socioeconomic inequalities, and family environments shape both obesity and mental health during adolescence. Such perspectives will contribute to the development of more effective, context-sensitive strategies for prevention and treatment, ultimately reducing the burden of both conditions at the population level.

## 5. Conclusions

The review confirms a significant association between obesity and mental health in children and adolescents, with depression being one of the most common manifestations. This relationship is complex and bidirectional, influenced by psychological, social, and biological factors, such as body dissatisfaction and social stigma. The evidence supports the adoption of integrated, multidisciplinary and individualized approach, integrating nutritional, psychological, social, and educational aspects, adapted to the characteristics and context of the individual. Prevention, early detection, and treatment strategies should reflect this dual approach to reduce the long-term burden of obesity and mental disorders. It is essential to incorporate systematic mental health assessments into programs targeting this population, prioritizing overall well-being over weight reduction alone. Finally, future research should focus on longitudinal studies with longer follow-up, comparisons between therapeutic interventions, and a more in-depth analysis of the psychological mechanisms linking obesity and mental health.

## Figures and Tables

**Table 1:** BMI classification in adult and pediatric populations.

BMI Classification	Adult Population (≥18 Years)	Pediatric Population (5–19 Years)
**Underweight**	<18.5	<−2 SD
**Normal weight**	18.5–24.9	≥−2 SD and ≤+1 SD
**Overweight**	25.0–29.9	>+1 SD and ≤+2 SD
**Obesity (Class I)**	30.0–34.9	>+2 SD and ≤+3 SD
**Obesity (Class II)**	35.0–39.9	>+3 SD
**Obesity (Class III)**	≥40.0	—

Source: Reference [1].

**Table 2:** Direct relationship between BMI and mental health.

Author (Year)	Sample	Study Design	Objective	Assessment Methods	Results	Conclusions	Limitations
**Ocampo et al., 2017** [36]	*N* = 180 (Females), 14–19 years	Cross-sectional	To determine the relationship between BMI and depressive symptoms in adolescent females.	- Depression: BDI-IA - BMI	25.56% presented depressive symptoms. Elevated BMI was associated with depression, relative risk 8.55 (*p* < 0.05).	Adolescents with overweight or obesity have higher risk of depressive symptoms, highlighting the need to address emotional factors in prevention.	Cross-sectional design, female-only sample, and self-reported measures, limiting generalizability and causal inference.
**Zhao et al., 2019 [30]**	*N* = 1081 (Males and Females), 10–12 years	Cross-sectional	To explore the relationship between obesity and depressive symptoms in Chinese adolescents, analyzing sex differences and specific depression dimensions.	- Depression: CES-D - BMI	Prevalence of depression 23.22%. Significant association between obesity and depression (OR = 1.47; 95% CI: 1.14–1.91; *p* = 0.004), stronger in males.	There is a significant relationship between obesity and depressive symptoms, with greater vulnerability in males. In males, several depressive aspects were associated with obesity, while in females only a lack of positive affect was associated with obesity.	Cross-sectional design that prevents establishing causality; self-reported BMI; possible bidirectionality between obesity and depression; no follow-up.
**Alsaleem et al., 2021 [42]**	*N* = 398 (Males), 14–19 years	Cross-sectional	To determine the relationship between obesity and depressive, anxiety, and stress symptoms in male adolescents in Saudi Arabia.	- Mental health: DASS-21 - BMI	65.7% of obese adolescents exhibited depressive symptoms. Obesity was significantly associated with depression (OR = 1.90), anxiety (OR = 1.97), and stress (OR = 1.89), *p* < 0.05.	Obesity in male adolescents is associated with higher prevalence of depression, anxiety, and stress.	Cross-sectional design, exclusively male sample, and reliance on self-reports limit generalizability and causal inference.
**Al Dweik et al., 2022 [46]**	*N* = 395 (Males and Females), 12–18 years	Prospective longitudinal	To examine the relationship between obesity, sleep duration, and depressive symptoms in adolescents in the UAE.	- Depression: MSPHQ - BMI	Significant positive correlation between BMI and depression levels (*p* < 0.001). Shorter sleep duration was associated with higher depression scores and higher BMI.	Obesity and insufficient sleep are linked to greater risk of depressive symptoms in adolescents.	Limited longitudinal design, self-reported measures, and no sex-specific analysis, limiting generalizability.
**Morán et al., 2024 [2]**	*N* = 397 (Males and Females), 10–19 years	Cross-sectional	To analyze the association between BMI, body dissatisfaction, self-esteem, and depressive symptoms in adolescents.	- Depression: BDI-II (>14 years), Birleson Children’s Depression Inventory (<14 years) - Body dissatisfaction: BSQ - Self-esteem: Rosenberg - BMI	Significant correlations were found between BMI z-score and depression (r = 0.36), body dissatisfaction (r = 0.56), and self-esteem (r = −0.42), all *p* < 0.001. Females exhibited higher levels of psychological distress.	Excess weight in adolescents is associated with greater psychological distress, particularly in females, and with poorer body image and self-esteem.	Cross-sectional design, self-reported measures, and sample limited to a single Chilean city, restricting generalizability.

**Table 3:** No direct relationship between BMI and mental health.

Author (Year)	Sample	Study Design	Objective	Assessment Methods	Results	Conclusions	Limitations
**Lim et al., 2016** [48]	*N* = 759 (Males and Females), 10–18 years	Prospective longitudinal	To identify factors associated with depressive symptoms in Korean primary, secondary, and high school students.	- Depression: CDI - Self-esteem: single-item question on feeling valued and respected - BMI	Prevalence of depressive symptoms: 11.5% primary, 11.4% secondary, 10.1% high school. Low self-esteem was the most associated factor (OR = 0.048–0.100; *p* < 0.001). In high school, being female (OR = 3.319; *p* = 0.001) was associated with higher risk.	Body perception and self-esteem are more relevant than BMI for depressive symptoms, especially in girls. School and family environment influence outcomes.	Loss to follow-up; no hormonal biomarkers included, limiting sex-specific analysis.
**Roberts & Duong, 2015** [15]	*N* = 4175 (Males and Females), 11–17 years	Prospective longitudinal	To evaluate whether body image mediates the relationship between obesity and depression and sex differences.	- Depression: DSM-IV - Body image: perceived weight - BMI	Major depression predicted future obesity only in adolescents perceiving themselves as overweight. Perceived overweight increased obesity risk 30-fold (40-fold in females). Adjusting for perception, the depression-obesity relationship disappeared (full mediation).	Negative body image explains the relationship between obesity and depression, especially in girls. Actual weight has less impact than body perception.	Not a national sample; BMI only used for adiposity; body image measured only via weight perception; no life-course data.
**Eitle & Eitle, 2018** [28]	*N* = 7461 (Males and Females), 12–18 years	Prospective longitudinal	To examine the relationship between overweight, perceived overweight, and depressive symptoms in adolescents.	- Depression: CES-D - BMI	No significant association was found between actual BMI and depressive symptoms. Perceived overweight was associated with higher depressive symptomatology, especially in African-American males.	Perceived overweight, more than objective weight, is related to greater depression in adolescents, highlighting the role of stigma and body image.	Self-reported data; limitations in establishing causality and bidirectionality; data are outdated.
**Carriere et al., 2019** [35]	*N* = 115 (Males and Females), 11–18 years	Cross-sectional	To explore the association between emotional disorders, personality dimensions, and binge eating disorder (BED) in obese adolescents.	- Depression: BDI - Anxiety: BAI - Eating behavior: BES - Alexithymia: TAS - Impulsivity: BIS - BMI	Adolescents with medium-high or high adiposity presented higher risk of depressive symptoms (OR = 1.38 and 1.59) and greater psychological distress (OR = 1.28 and 1.62) versus stable-normal weight. They reported lower mental well-being.	BMI is associated with BED, and depression is also associated with BED; however, no direct association between BMI and depression was found.	Use of self-reports, potential recall bias, and lack of control for psychosocial factors affecting mental health.
**Lima et al., 2020** [27]	*N* = 1296 (Males and Females), 15 years	Cross-sectional	To investigate the relationship between social anxiety, depression, and abdominal obesity, analyzing sex differences.	- Depression: CES-D - Social anxiety: SAS-A - Waist circumference	Females showed higher social anxiety and depression (*p* < 0.001). Social anxiety was associated with greater waist circumference (β = 0.08; *p* = 0.005) and depressive symptoms (β = 0.42; *p* < 0.001). Depression was not associated with obesity nor acted as a mediator.	Female adolescents present higher social anxiety and depression. Social anxiety is related to obesity, but depression has no direct association with waist circumference or mediation.	Cross-sectional design limits causality; sample from one region in Brazil; only social anxiety evaluated.
**Wang et al., 2020** [37]	*N* = 153 (Males and Females), 12–13 years	Prospective longitudinal	To evaluate body dissatisfaction and depressive symptoms by weight and bidirectional relationships in Black adolescents.	- Depression: BDI-I - Body dissatisfaction: silhouette scale - BMI	In overweight/obese adolescents, body dissatisfaction predicted future depressive symptoms (β = 0.42; *p* < 0.001), and depression predicted higher body dissatisfaction (β = 0.25; *p* = 0.012). No effect was observed in healthy-weight adolescents.	Body dissatisfaction and depression are bidirectionally related in overweight adolescents, reflecting stigma and negative self-evaluation.	Small sample for sex differences; high proportion of low-income families; possible bias using BDI instead of CDI; non-causal design.
**López et al., 2021** [29]	*N* = 76 (Males and Females), 14–18 years	Prospective longitudinal	To analyze demographic, psychosocial, and intervention-type factors predicting participation in overweight/obese adolescents in treatment.	- Depression: CES-DC - Executive function: BRIEF-2 - BMI	No significant relationship was found between depressive symptoms or executive function and treatment adherence. Over 25% had moderate/severe depression at baseline. No sex differences observed.	Individual characteristics do not predict participation; family support appears key for treatment engagement.	Small sample size; potential uncontrolled variables; results not generalizable to adolescents not seeking treatment.
**Öz & Kıvrak, 2023** [16]	*N* = 98 (Males and Females), 11–17 years	Cross-sectional	To evaluate the impact of obesity on anxiety, depression, self-esteem, and emotional regulation in children and adolescents.	- Depression and anxiety: RCADS - Self-esteem: Rosenberg - Emotional regulation: DERS - Self-concept: PHCSCS - BMI	Significant differences (*p* < 0.05) were observed in anxiety, depression, emotional regulation, and self-esteem between groups	Obesity in children and adolescents is associated with increased anxiety, depression, low self-esteem, and difficulties in emotional regulation, which can negatively affect treatment. A relationship was also identified between poor regulation and emotional eating, with no significant differences by gender, age, or BMI.	

**Table 4:** Effectiveness of cognitive–behavioral interventions in adolescents with obesity.

Author (Year)	Sample	Design	Objective	Instruments	Main Results	Conclusions	Limitations
**Hoare et al., 2016** [68]	*N* = 634 (13–15 years, Males and Females)	Prospective longitudinal	To analyze association between lifestyle, obesity, and depressive symptoms	SMFQ, ABAKQ, BMI	Physical activity was associated with lower depression in males. Poor diet was associated with higher symptoms in females.	A healthy lifestyle is linked to better mental health in adolescents with obesity.	Use of self-reports, no control group, high non-participation rate.
**Naar-King et al., 2016** [67]	*N* = 186 (12–16 years, Males and Females)	Prospective longitudinal	To develop an adaptive behavioral treatment for African-American adolescents with obesity	CDI, BMI	Weight reduction (2.96%) without significant changes in depression. Better outcomes in adolescents with higher executive function.	Intervention reduced overweight, without clear impact on depression.	Small sample, self-reported data, need for further analyses.
**Nobles et al., 2016** [60]	*N* = 435 (10–15 years, Males and Females)	Retrospective longitudinal	To evaluate effectiveness of the SHINE program on BMI, mental health, and adherence	BMI, Self-esteem (Rosenberg), Anxiety, Depression	Sustained BMI reduction and mental health improvements up to 12 months. 54% retention.	SHINE program demonstrated effectiveness in reducing BMI and improving psychological well-being.	No control group, limited follow-up and adherence data.
**Gulley et al., 2019** [53]	*N* = 119 (12–17 years, M)	Prospective longitudinal	To evaluate whether reduction of depressive symptoms via CBT influences BMI and metabolic markers	CES-D, BMI	CBT did not directly reduce BMI, but decreases in depressive symptoms were associated with greater BMI reduction (B = 0.05).	CBT improves mental health and may influence BMI when depression is severe.	Limited sample size, small effects, risk of type I error, restricted generalizability.
**Tronieri et al., 2019** [56]	*N* = 7 (12–17 years)	Pilot, longitudinal	To evaluate feasibility of ACT + lifestyle change	PHQ-9, PSS-14, EDI-3, IWQOL-Kids, BMI	High acceptance; reductions in BMI and improvements in depressive symptoms, body image, and quality of life.	ACT combined with lifestyle changes is promising for adolescents with obesity.	No control group, very small sample, need for adaptation of techniques.
**Ramalho et al., 2020** [57]	*N* = 210 (13–18 years, Males and Females)	Prospective longitudinal	To evaluate effectiveness of online CBT intervention in Portuguese adolescents	DASS-21, ChEAT, BMI	High comorbidity between psychological symptoms and obesity, especially in females. Significant correlations between distress and quality of life.	Intervention relevant for assessing gender disparities and mental health.	Convenience sample, self-report bias, cross-sectional design.
**Tulloch et al., 2020** [59]	*N* = 228 (14–18 years)	Prospective longitudinal	To analyze relationship between depressive symptoms, stress, self-efficacy, and physical fitness	CDI, PSS, BMI	Self-efficacy predicted better aerobic fitness. Initial depression was associated with lower performance, especially in females.	Psychological factors influence physical performance in adolescents with obesity.	Gender-imbalanced, low diversity, limitations in exercise assessment.
**Darling et al., 2021** [63]	*N* = 66 (13–17 years, Males and Females)	Prospective longitudinal	To evaluate changes in depression and eating behavior after behavioral intervention	CDI-2, EDE-Q, BMI	No overall changes, but weight and shape concerns decreased in females.	Positive effects on body image without worsening mental health.	Small sample, no control group, no analysis by gender identity.
**Lemstra & Rogers, 2022** [54]	*N* = 2292 (11–17 years, Males and Females)	Prospective longitudinal	To evaluate a free community program for mood improvement	CES-D, NLSCY, SF-12, BMI	Significant reduction in depressive symptoms (d = 0.94) at 12 weeks and 1 year.	Community intervention effective for improving mental health in youth with obesity.	No control group or randomization; results show associations, not causality.

**Table 5:** Effects of restrictive dietary therapy in adolescents with obesity.

Author (Year)	Sample	Study Design	Objective	Assessment Instruments	Main Results	Conclusions	Limitations
**Jebeile et al., 2024** [55]	*N* = 141 (13–17 yrs)	Prospective longitudinal trial	To evaluate changes in depressive symptoms, eating disorders, and binge eating during the Fast Track to Health program	CESDR-10, EDE-Q, BES, BMI	Significant reduction in depressive symptoms (22% to 9%) and eating disorder symptoms (110 to 56 participants) over 52 weeks	Dietary intervention showed positive effects on mental health and eating behaviors in adolescents with obesity; some required additional follow-up	Self-reports, possible overlap between EDE-Q subscales and treatment content, influence of COVID-19 pandemic context

**Table 6:** Effects of surgical treatment on adolescents with obesity.

Author (Year)	Sample	Study Design	Objective	Assessment Instruments	Main Results	Conclusions	Limitations
**Järvholm et al., 2015** [61]	*N* = 88 (13–18 yrs, M/F)	Prospective longitudinal study	To evaluate mental health changes over 2 years in adolescents undergoing gastric bypass	BYI, BDI-2, Rosenberg Self-Esteem, BMI	Significant reductions in anxiety, depression, anger, and disruptive behavior (*p* = 0.001–0.022), with improved self-esteem and mood (*p* < 0.001–0.025), especially during the first year. At 2 years, 19% still had clinical depressive symptoms	Gastric bypass significantly improves mental health in adolescents with obesity, but a considerable proportion requires ongoing psychological follow-up	No control group, reliance on self-reports, assessment norms not adjusted for >18 yrs, possibly underestimating persistent psychological issues

## Data Availability

No new data were created or analyzed in this study.
